# Mitogenomics provides new insights into the phylogenetic relationships and evolutionary history of deep-sea sea stars (Asteroidea)

**DOI:** 10.1038/s41598-022-08644-9

**Published:** 2022-03-18

**Authors:** Shao’e Sun, Ning Xiao, Zhongli Sha

**Affiliations:** 1 Department of Marine Organism Taxonomy and Phylogeny, Institute of Oceanology, Chinese Academy of Sciences, Nanhai Road 7, Qingdao, 266071 China; 2 Laboratory for Marine Biology and Biotechnology, Qingdao National Laboratory for Marine Science and Technology, Qingdao, 266071 China; 3 Shandong Province Key Laboratory of Experimental Marine Biology, Institute of Oceanology, Chinese Academy of Sciences, Qingdao, 266071 China; 4 University of Chinese Academy of Sciences, Beijing, 100049 China

**Keywords:** Evolution, Genetics, Molecular biology

## Abstract

The deep sea (> 200 m) is considered as the largest and most remote biome, which characterized by low temperatures, low oxygen level, scarce food, constant darkness, and high hydrostatic pressure. The sea stars (class Asteroidea) are ecologically important and diverse echinoderms in all of the world’s oceans, occurring from the intertidal to the abyssal zone (to about 6000 m). To date, the phylogeny of the sea stars and the relationships of deep-sea and shallow water groups have not yet been fully resolved. Here, we recovered five mitochondrial genomes of deep-sea asteroids. The A+T content of the mtDNA in deep-sea asteroids were significantly higher than that of the shallow-water groups. The gene orders of the five new mitogenomes were identical to that of other asteroids. The phylogenetic analysis showed that the orders Valvatida, Paxillosida, Forcipulatida are paraphyletic. Velatida was the sister order of all the others and then the cladeValvatida-Spinulosida-Paxillosida-Notomyotida versus Forcipulatida-Brisingida. Deep-sea asteroids were nested in different lineages, instead of a well-supported clade. The tropical Western Pacific was suggested as the original area of asteroids, and the temperate water was initially colonized with asteroids by the migration events from the tropical and cold water. The time-calibrated phylogeny showed that Asteroidea originated during Devonian-Carboniferous boundary and the major lineages of Asteroidea originated during Permian–Triassic boundary. The divergence between the deep-sea and shallow-water asteroids coincided approximately with the Triassic-Jurassic extinction. Total 29 positively selected sites were detected in fifteen mitochondrial genes of five deep-sea lineages, implying a link between deep-sea adaption and mitochondrial molecular biology in asteroids.

## Introduction

The Asteroidea (sea star) is the second most diverse class of echinoderms after the Ophiuroidea (~ 2100 species), with approximately 1900 accepted extant species worldwide, grouped into 36 families 370 extant genera^1,2^. Asteroids are widely spread in all of the world’s oceans, but reveal highest levels of species richness in the Indo-Pacific and tropical Atlantic regions^1,3–5^. They present at all depths from the shallow-water to the deep sea (~ 6000 m)^1,6^. Out of the 36 families of living Asteroidea, 15 of those occur exclusively in deep-sea settings and 14 families have deep-sea members^1^. However, to date, the relationships of the starfish have not yet been fully resolved^1,7–10^. The monophyly of the order Valvatida still not supported^7,8,10^. Another most important issue in asteroid phylogeny is the phylogenetic position of the order Paxillosida. Two contrasting hypotheses have been proposed with regard to the phylogenetic position of Paxillosida. Some morphological and molecular-based evidences supported that the Paxillosida was a primitive asteroid group^11–19^, as they lacks an anus and suckers on the tube feet. However, an opposite opinion argued that the paxillosidans diverged later during asteroid evolution^1,8,10,20–28^. The controversy highlights the need for a well sampled and well-supported phylogenetic relationship for asteroids, to distinguish the early divergence asteroid orders and locate the problematic and morphologically unusual taxa.

The deep sea (> 200 m), which is considered as the largest and most remote biome of the world, occupyed about 66% of the bottom of the global ocean^29^. The deep sea was characterized by low temperatures, high hydrostatic pressure, low oxygen level, food supply scarcity and constant darkness^30,31^. If the marine invertebrates originated in shallow-water or the deep sea have been debated for a long time. The studies of different taxa, e.g. molluscan, crustacean, corals and holothurians, have revealed a variety of different relationships. An analysis of the deep-sea molluscan distribution and taxonomy revealed that the deep sea was initially colonized with molluscans by migration events from the shallow water^32^. The phylogenetic trees inferred with respectively the mitogenomes, the nuclear, and the combined datasets suggested that multiple transitions between shallow water and deep-sea habitats occurred during the evolution of the vetigastropods molluscan^33^. Using complete 18S and partial 28S rDNA, Raupach et al.^34^ found that the deep-sea asellote isopods probably shaped by invasions from adjacent shallow-water regions^34^. The shallow to deep pattern has also been proposed to explain the evolution of deep-sea decapod crustaceans based on mitogenomes^35^. Lindner et al.^35^ found the stylasterid corals originated and diversified extensively in the deep sea, and subsequently invaded shallow waters^36^. The mitogenomic phylogenetic relationship of the holothurians showed that the deep-sea species formed the basal clades in the phylogenetic trees^37^.

The mitochondrial genomes (mitogenomes) are characterized by maternal inheritance, small genome size, fast nucleotide substitution rate and high mutation rate^38,39^. Therefore, mitochondrial DNA (mtDNA) is widely used as an informative molecular marker for study of phylogenetic relationships^40–42^, phylogeography^43^ and genomic evolution^44–46^. Furthermore, the complete mitogenomes show greater accuracy than single mtDNA markers, and could obtain more accurate phylogenetic relationships^47,48^. In addition, all the 13 mitochondrial PCGs that involved in oxidative phosphorylation play key roles in oxygen usage and energy metabolism. Despite strong functional constraints, mtDNA may evolve under positively selection in response to pressures from extreme harsh environment^49^. Variation in mitochondrial mtDNA can directly influence metabolic performance, an increasing number of studies related to adaptive evolution of mtDNA have been reported^35,50–52^.

Prior to our study, only twenty-five mitogenomes from Asteroidea are available, which distributed within fourteen families (Acanthasteridae, Archasteridae, Asterinidae, Asteriidae, Astropectinidae, Echinasteridae, Freyellidae, Goniasteridae, Luidiidae, Ophidiasteridae, Oreasteridae, Pterasteridae, Porcellanasteridae, Solasteridae). Compared to their diversity and abundance, the mitogenome sequences are still limited in the class Asteroidea. Among all available mitogenomes of asteroids, data from deep-sea environments are so scarce that only two mitogenomes have been reported to date^53,54^. The studies on them would provide statistically meaningful data for various aspects, such as origin and evolution and even adaptive mechanisms in deep-sea extreme environments.

In this study, we newly sequenced and annotated five complete mitogenomes of the deep-sea sea stars (*Cheiraster* sp. Studer, 1883, *Zoroaster ophiactis* Fisher, 1916, *Brisinga* sp. Asbjørnsen, 1856, *Paulasterias* sp. Mah et al.^55^, *Asthenactis papyraceus* Fisher, 1916) from the families Benthopectinidae, Zoroasteridae, Brisingidae, Paulasteriidae, and Myxasteridae, respectively. Currently, no mitogenome has been reported in these families. This paper aims to improve our understanding of the higher-order phylogeny and evolution history of deep-sea asteroid by the following: (1) comparing the deep-sea asteroid mitogenomes (the bias of strand nucleotide composition and the codon usage) with that from shallower habitats; (2) evaluating the phylogenetic relationships and estimating the divergence time of asteroids; (3) reconstructing the ancestral geographic area and habitat of asteroids; (4) estimating the selective pressures operating on the deep-sea asteroids mitogenomes in order to understand the genetic basis of deep-sea adaptation in sea stars.

## Results and discussion

### Mitochondrial genome assemblies and organization

The sequencing output data for the five species of deep-sea sea stars were summarized in Table 1. There were about 4.07–8.38 Gb clean reads, 97.33–98.50% of the reads passed Q20. The mitogenomes of the five asteroids were all circularized using NOVOPlasty from the reads, with 16,042 (*Paulasterias* sp.) to 16,426 bp (*Cheiraster* sp.) in length. The size range primarily due to the size variation of intergenic regions. The annotations, length and strand position of all PCGs and RNA genes of the five mitogenomes were summarized in Supplementary Table S1. For all the five mitogenomes, 37 genes (13 protein-coding genes (PCGs), 2 ribosomal RNA genes (rRNAs) and 22 transfer RNA genes (tRNAs)) were detected as typical in other metazoans^56^. Within these genes, *nad1*, *nad2*, *nad6*, *rrnL* and eleven tRNA genes (tRNA-Ser^UCN^, tRNA-Ile, tRNA-Leu^UUR^, tRNA-Gly, tRNA-Tyr, tRNA-Met, tRNA-Cys, tRNA-Trp, tRNA-Leu^CUN^, tRNA-Asn, tRNA-Pro) were encoded by the minority strand, and the remaining ones were encoded by the major strand. The gene order of the five new mitogenomes were identical to that of other asteroids.

**Table 1:** New mitochondrial genomes analyzed in present study.

Species	Genbank accession no	Mitogenome size (bp)	Clean data (Mb)	Clean data Q20 (%)	Location	Positioning	Coordinates	Depth (m)	Cruise details	Collect time
*Cheiraster* sp.	MZ702701	16,426	8,581.0	97.58	Tropical Western Pacific Ocean	FX-Dive219	10.1114 N, 140.2422E	899	Remotely Operated Vehicle (ROV)	06/07/19
*Paulasterias* sp.	MZ702702	16,042	4,938.9	97.82	Tropical Western Pacific Ocean	FX-Dive222	10.0781 N, 140.1519E	2141	Remotely Operated Vehicle (ROV)	06/10/19
*Asthenactis papyraceus*	MZ702703	16,076	4,718.7	97.33	Tropical Western Pacific Ocean	FX-Dive63	11.2726 N, 139.3702E	1569	Remotely Operated Vehicle (ROV)	03/21/16
*Zoroaster ophiactis*	MZ702704	16,193	4,164.7	98.28	Tropical Western Pacific Ocean	FX-Dive16	8.8608 N, 137.7344E	1108	Remotely Operated Vehicle (ROV)	12/15/14
*Brisinga* sp.	MZ702705	16,147	6,988.6	98.50	Tropical Western Pacific Ocean	FX-Dive63	11.2830 N, 139.3770E	1126	Remotely Operated Vehicle (ROV)	03/21/16

For all the five species, most PCGs start with the codon ATN. Deviations occurred in *nad1* (*Z. ophiactis* and *Brisinga* sp.) and *nad2* (*Paulasterias* sp. and *Z. ophiactis*), which started with GTG. Most PCGs were stopped by the complete stop codons (TAG and TAA), but in *Cheiraster* sp. and *A. papyraceus* the *cox2* gene, and in *Z. ophiactis*, *A. papyraceus* and *Paulasterias* sp. the *cytb* gene were ended with a single T nucleotide. The *cytb* gene in *Brisinga* sp. and *Cheiraster* sp. were ended with TA. The gene orders of the five new asteroids mitogenomes were identical to these of other sea stars, but distinct from the echinoderm ground pattern^52^.

### Base composition and codon usage of mtDNA in Asteroidea

The five new mitogenomes were AT rich (> 60%), with *A. papyraceus* having the highest (68.30%) and *Paulasterias* sp. having the lowest (62.94%) AT content. Among the reported asteroids mitogenomes, the highest A+T content 68.07%, 68.23% and 68.30% occurred in the deep-sea species *F. benthophila* (MG563681), *Brisinga* sp. and *A. papyraceus*, respectively. In order to explore if the A+T content between the deep-sea and shallow-water species was different, the A+T content at complete mtDNA, PCG, tRNA, and rRNA genes were compared. Statistical *t*-tests showed that the A+T content of the complete mtDNA (two-tailed *t*-test, *p* = 0.007), PCG (*p* = 0.012), tRNA (*p* = 0.001), and rRNA (*p* = 0.012) in deep-sea asteroids were significantly higher than those of the shallow-water species (Supplementary Table S2). This phenomenon highlights the divergence of base composition between deep-sea and shallow water asteroids.

Among PCGs, the Leucine (15.40–16.70%) and Serine (9.11–10.66%) were the most frequently used amino acids, whereas Cysteine (0.86–1.21%), Arginine (1.65–2.04%) and Aspartic Acid (1.50–1.88%) were rarely used ones (Supplementary Table S3). As all the 13 mitochondrial PCGs were transmembrane proteins^57^, and the membrane-based processes were sensitive to the lowered temperature and increased hydrostatic pressure of deep sea^58^. Considering that the amino acid composition and properties may influence the protein functions^59–61^, we compared these two parameters between the deep-sea and shallow water asteroids. However, we failed to find significant differences in the percentages of non-polar and polar-uncharged, as well as the charged amino acids between the deep-sea and shallow water groups, which may be owing to the bias of sample sizes.

**Figure 1:**
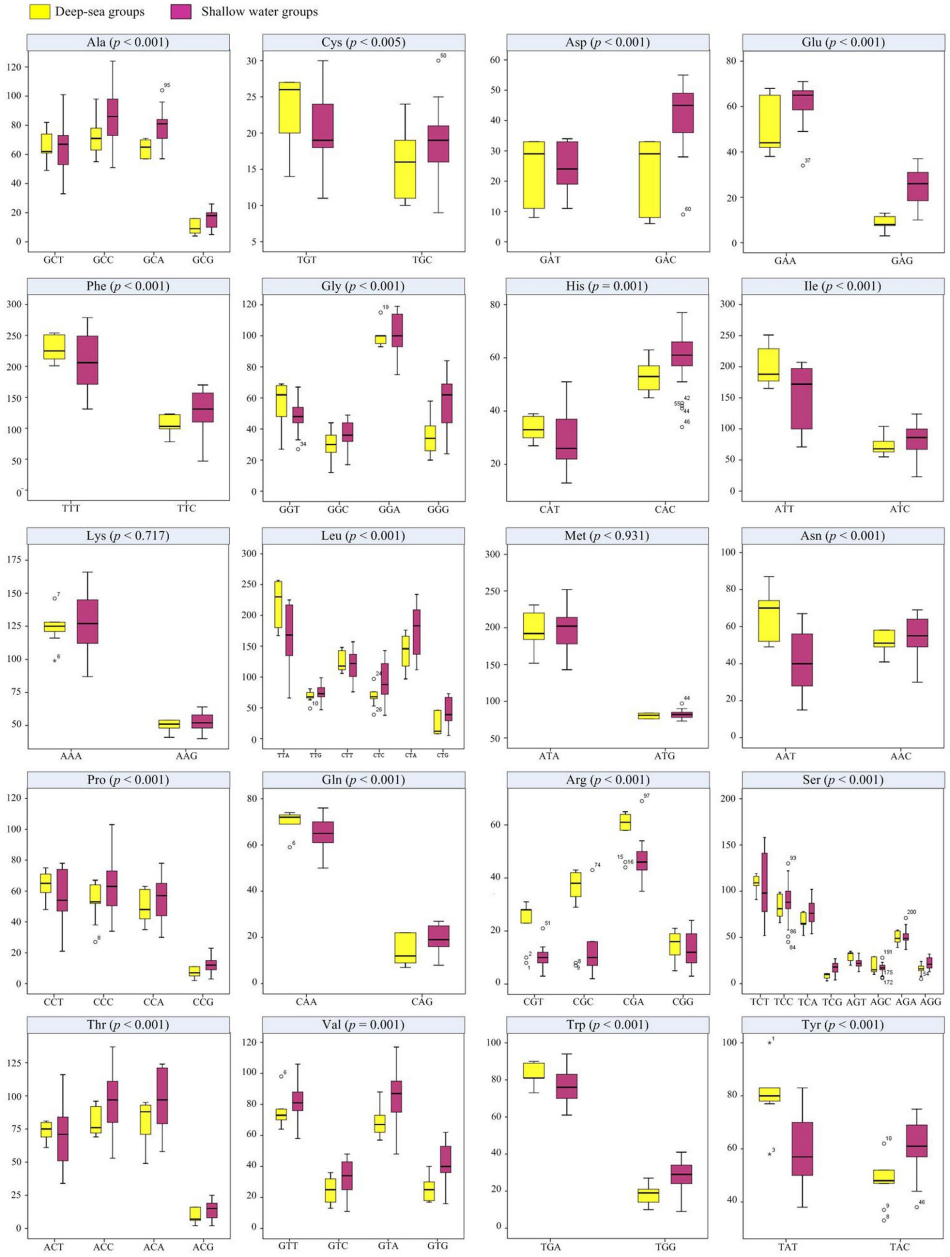
Comparisons of codon usage (y-axis) between the deep-sea and shallow water asteroids. x-axis indicated the synonymous codons. The amino acid and its corresponding *p*-value were shown at the top of each box plot.

Figure 1 showed the strong A+T bias in codon usage across 30 asteroids mitogenomes. Among the 62 available codons, the six most used codons were TTT (Phe), TTA (Leu), CTA (Leu), ATT (Ile), ATA (Met) and AAA (Lys), almost all of them made up of solely A and T nucleotides. Even so, in the deep-sea species, the preference for A+T codons was stronger than the shallow water group. Statistical *t*-tests showed that for every amino acid, the proportions of the codons were significantly different between the deep-sea and shallow water asteroids (*p*-values in Fig. 1). The evolution of eukaryotic genome demonstrates a universal mutational bias toward A+T composition, leading to the hypothesis that genome-wide nucleotide composition generally evolves to a point at which the selective power in favor of G+C is approximately balanced by the power of random genetic drift, therefore variation in equilibrium genome-wide nucleotide composition is largely defined by variation in mutation biases^62^. Based on this hypothesis, the differences in nucleotide composition between the deep-sea and shallow-sea asteroids not only have functional implications, but also evolved as a result of a random genetic drift. In addition, the vast majority of the structures and components of the mitochondrion is encoded by nuclear genes^63^. This means that the functions of every product from mitogenome are association with products of the nuclear genome^64,65^. In other words, nuclear genome DNA may be a potential driver for mtDNA differences between the deep-sea and shallow-sea asteroids.

### Phylogenetic analysis

**Figure 2:**
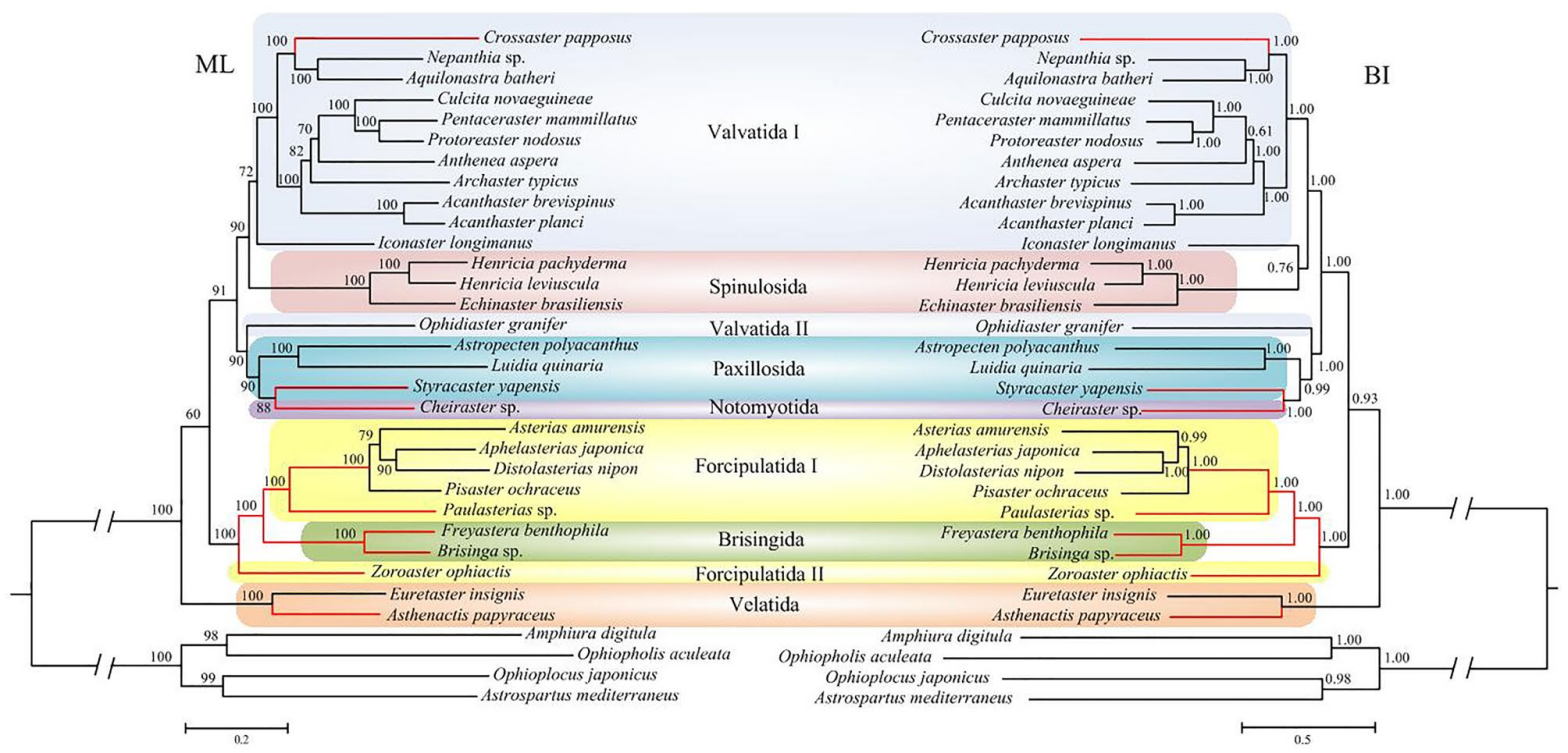
Phylogenetic analysis of ML and BI based on the nucleotide sequencces of the 13 concatenated protein-coding genes. The bootstrap probability and the Bayesian posterior probability were shown at each node. The deep-sea branchs were marked by red.

**Figure 3:**
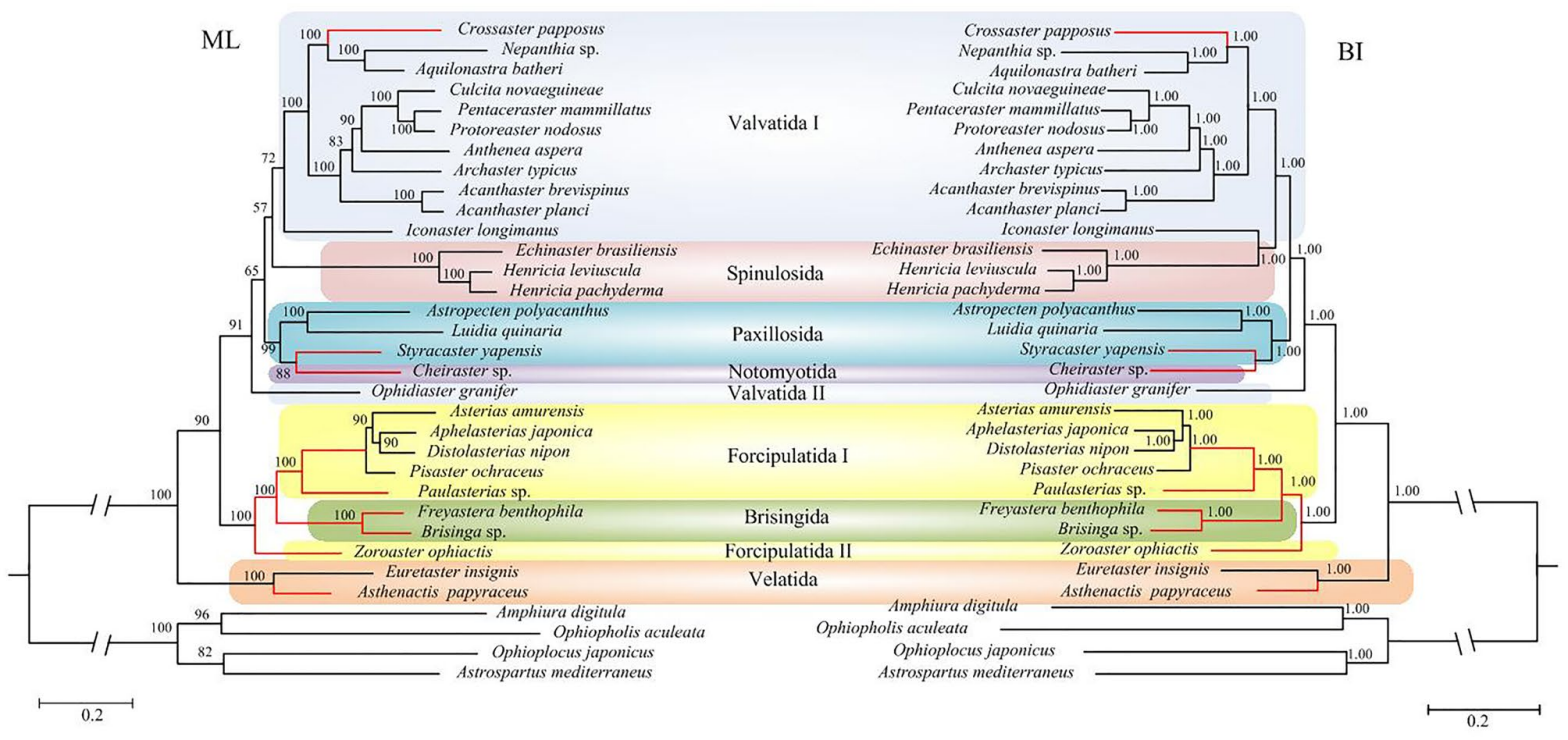
Phylogenetic analysis of ML and BI based on the amino acid sequencces of the 13 concatenated protein-coding genes. The bootstrap probability and the Bayesian posterior probability were shown at each node. The deep-sea branchs were marked by red.

Although previous studies investigated the relationships within the class Asteroidea using molecular data^1,7–10,66^, the relationships has remained controversial up to now, with no consensus emerged. Our trees represented one of the well-sampled (for both taxa and molecular marker) and well-supported phylogenomic evidences for asteroid phylogeny. In this study, the phylogenetic analyses (ML and BI) were performed for the 30 species of sea stars based on the nucleotide (Fig. 2) and amino acid sequences (Fig. 3) of 13 mitochondrial PCGs. All the analysis produced phylogenies that differed only slightly. These topologies recovered that deep-sea asteroids were nested in different lineages, instead of a well-supported clade. Velatida was the sister Order of all the others and then the clade Valvatida-Spinulosida-Paxillosida-Notomyotida versus Forcipulatida-Brisingida. Our study suggested monophyly only for four asteroid orders, Spinulosida, Notomyotida, Brisingida and Velatida. However, the phylogenetic analysis revealed several discrepancies between our molecular results and current taxonomy at order levels. The unwell-supported clades included Valvatida, Paxillosida, Forcipulatida. These results were not perfectly consistent with the previous works based on transcriptomics datasets^9,10^, and limited molecular markers^1,7–9^. This discrepancy may due to the heterogeneity of data. These results were also different from the recent mitogenomic studies of Asteroidea in some orders^66^, which contained limited taxonomic sampling. Some primarily deep-sea taxa form orders Brisingida, Forcipulatida and Notomyotida have not been sampled in study of Zheng et al.^66^.

The Valvatida, the most diversified Order within the Asteroidea, was never recovered as monophyletic in any of previous studies^1,10^. Our results concerning the taxonomic relationships within Valvatida are sensitive to the choice of dataset and analytical methods. Ophidiasteridae (n = 106) is the largest asteroid family throughout the tropical shallow-water Atlantic, and Indo-Pacific^1,4^. Our phylogenetic trees based on nucleotide sequence showed that the Ophidiasteridae (represented by *Ophidiaster granifer*) was placed in a branch (Valvatida II) as a sister group to Paxillosida + Notomyotida, then grouped with Valvatida I + Spinulosida (Fig. 2). However, the amino acid sequences based trees supported Ophidiasteridae (Valvatida II) as a sister clade to Valvatida I + Spinulosida + Paxillosida + Notomyotida (Fig. 3). The family Goniasteridae (n = 256) contained the largest number of species within the Asteroidea^1^. In the ML trees, Goniasteridae (represented by *Iconaster longimanus*) was the sister family to the remaining families of Valvatida (except Ophidiasteridae) (Fig. 2). While in the BI trees, Goniasteridae was more closely related to Spinulosida, instead of other species of Valvatida (Fig. 3). Thus, the Valvatida is paraphyletic, and the phylogenetic positions of Ophidiasteridae and Goniasteridae are still need to be resolved.

As summarized in the introduction, the phylogenetic status of the order Paxillosida, primitive or derived, is a controversial issue in asteroid phylogeny^67^. Our trees showed that the Paxillosida + Notomyotida lineages later diverged within the Valvatida + Spinulosida + Paxillosida + Notomyotida clade (Figs. 2, 3). This result was consistent with a “Paxillosida is derived” perspective^1,8,10,20–28^, which countered the “Paxillosida is primitive” scenario^11–19^. In present study, the deep-sea paxillosidan *Styracaster yapensis* and notomyotidan *Cheiraster* sp. formed a well-supported clade, making the Paxillosida paraphyletic. This relationship has never been observed in prior literature^10^, which may benefited from the well-sampled asteroid taxa in our study.

An earlier cladogenetic event among extant asteroids separated Forcipulatida + Brisingida from the Valvatida + Spinulosida + Paxillosida + Notomyotida clade (Figs. 2, 3). The close relationship between the Forcipulatida and Brisingida has also been suggested by Gale^15^, Knott and Wray^7^, Linchangco et al.^10^, Zheng et al.^66^. In this study, the deep-sea brisingidans *Freyastera benthophila* and *Brisinga* sp. nested within Forcipulatida, and closely related with the Forcipulatida species (Forcipulatida I in Figs. 2, 3). Thus, the monophyletic of Forcipulatida was not supported. Our results were similar to those of Knott and Wray^7^. The family Zoroasteridae is a small but widespread group of forcipulatidan asteroids distributed throughout the deep sea from bathyal to abyssal habitats (200–6000 m)^6^. In our trees, the Zoroasteridae (represented by *Zoroaster ophiactis*) was recovered as the basal forcipulatidan lineage (Forcipulatida II), as suggested by Knott and Wray^7^ and Mah and Foltz^68^. The Paulasteriidae is a new family described from deep-sea settings, representing the first new taxon within the Forcipulatacea since the 1800s^55^. Based on the molecular evidence presented in Figs. 2, 3, the deep-sea species *Paulasterias* sp. from the Paulasteriidae showed the closest relationship with the species from Forcipulatida, which is consistent with the phylogenetic status revealed by three-gene data set (12S rDNA, 16S rDNA, and histone H3 genes)^55^.

Velatida was represented here by two species from the family Pterasteridae and the deep-sea Myxasteridae that formed a clade, occupying a basal position separated from other asteroids (Figs. 2, 3). This is consistent with the research of Janies et al.^8^, placing the Velatida as the earliest branch in the evolution of Asteroidea. However, our study differs from the previous placement of Velatida as a sister group to Forcipulatida^10^, and sister to Forcipulatida+Brisingida^66^. So a more representative sampling is in need to better assess the relationships among the asteroid orders.

### Geographical and habitat origin of the Asteroidea

**Figure 4:**
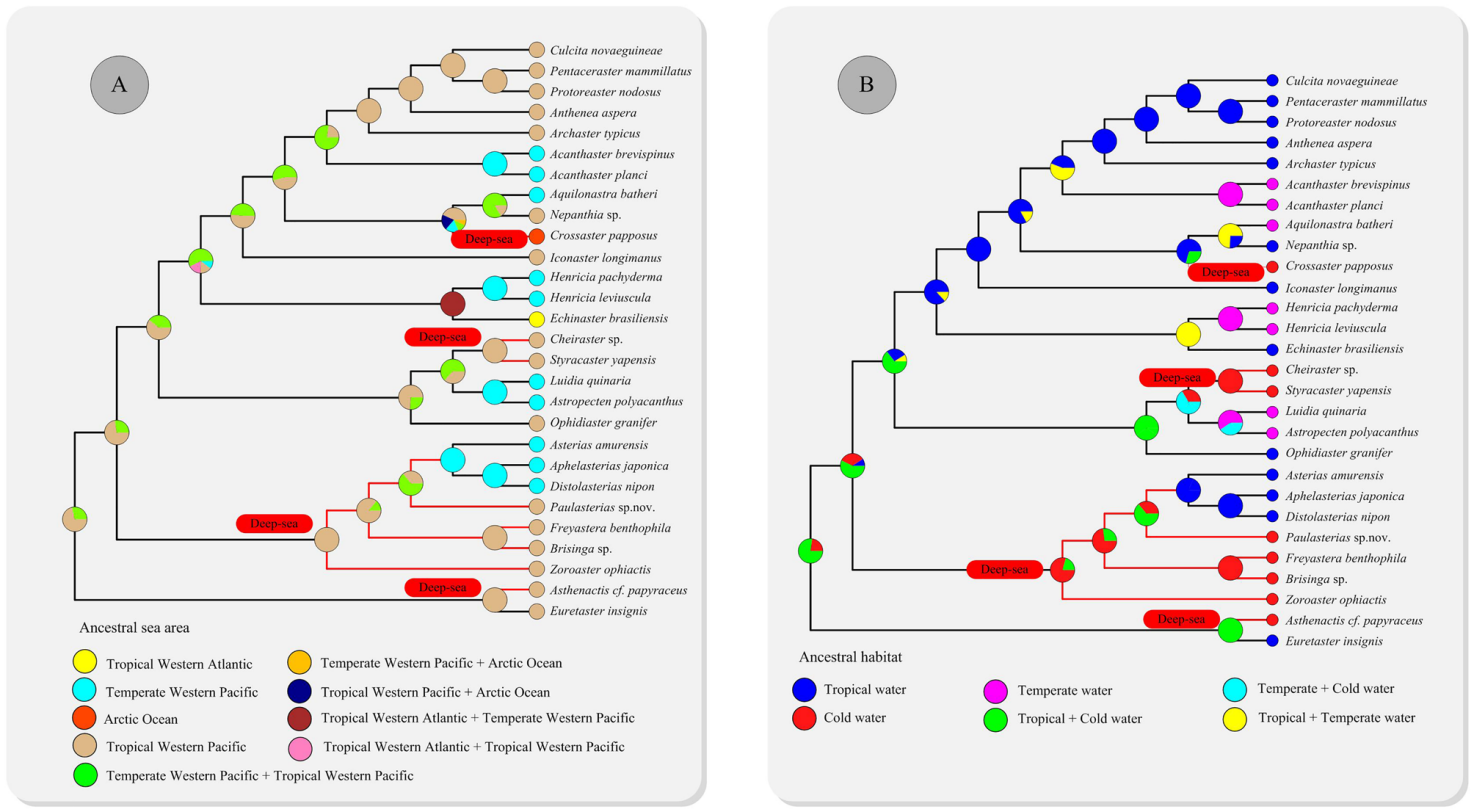
Historical biogeography (**A**) and habitat (**B**) of Asteroidea. The scenario was inferred from three different models (S-DIVA, S-DEC and DEC) based on the ML tree constructed in our study. Pie charts near nodes indicated the probabilities of certain ancestral geographic areas and habitat. Red labels indicated the deep-sea species.

Asteroids widely distribute in all of the world’s oceans and present at all depths from the intertidal to the abyssal (to about 6000 m)^1,3^. Combining the phylogenetic analyses with the ancestral geographic area can provide new insights into early geographical origin of the Asteroidea. The ancestral geographic area reconstruction provided evidences that the Most Recent Common Ancestor (MRCA) of asteroids may occurred in tropical Western Pacific, and the Western-Pacific ancestor results in seven difierent lineages invading the temperate Western Pacific, tropical Western Atlantic and Arctic regions (Fig. 4A). It is intriguing to note that the clades of the tropical Western Pacific were distributed with tropical and cold asteroids from both shallow water and deep-sea. The Western Pacific is also the source of diversity for asteroids^3–5^. As the main component of the modern Indo-Australian Archipelago (IAA), the Western Pacific is a modern hotspot of marine biodiversity^69^. Based on the species-energy framework, thermal energy shape the marine shallow water biodiversity, while chemical energy availability (export productivity) and connectivity to shallower habitats drive deep-sea diversity^6^. The IAA hotspot lies between 30° N and 30° S latitudes, with strong thermal energy input from the sun. Thus, higher seasonal surface productivity exist in it’s shelf and slope regions^6^. The radiation hypothesis also suggested that deep-water richness is maintained by immigration from shallower regions^69^. The IAA hotspot locates in the region of convergence between Eurasia, Australia, and the Pacific-Philippine Sea plates, where exist a complex mosaic of shallow-seas, arc, and microcontinental fragments^6^. Thus, the IAA region was marked by higher export productivity and proximity to shallower communities, which were the unique geographical and biological advantages to maintain high biodiversity in the deep sea.

The asteroids were broadly categorized as occurring in “cold”, “temperate”, and “tropical” based on sea-surface temperatures^1,70^. Generally, deep-sea and high-latitude settings are treated as “cold” temperatures^1,70^. In our study, the “cold” temperature mainly refers to the deep-sea settings, as only one high-latitude clade included here. The ancestral habitat reconstruction suggested that the temperate water was initially colonized with asteroids by the migration events from the tropical and cold water (Fig. 4B). Controversial hypotheses have been proposed to describe the evolutionary origin for the marine invertebrates, e.g. shallow-water origin^32,35,71–73^, deep-sea origin^36^ or in both directions^74,75^. The new results presented herein was sufficient to illustrate that, not conclusive, the deep sea setting was an ancestral habitat of the asteroids. Our recent evolutionary study of Holothuroidea (sea cucumbers) revealed a similar phenomenon^37^. More samples are required to clarify their origin and evolution.

### Divergence time estimation

**Figure 5:**
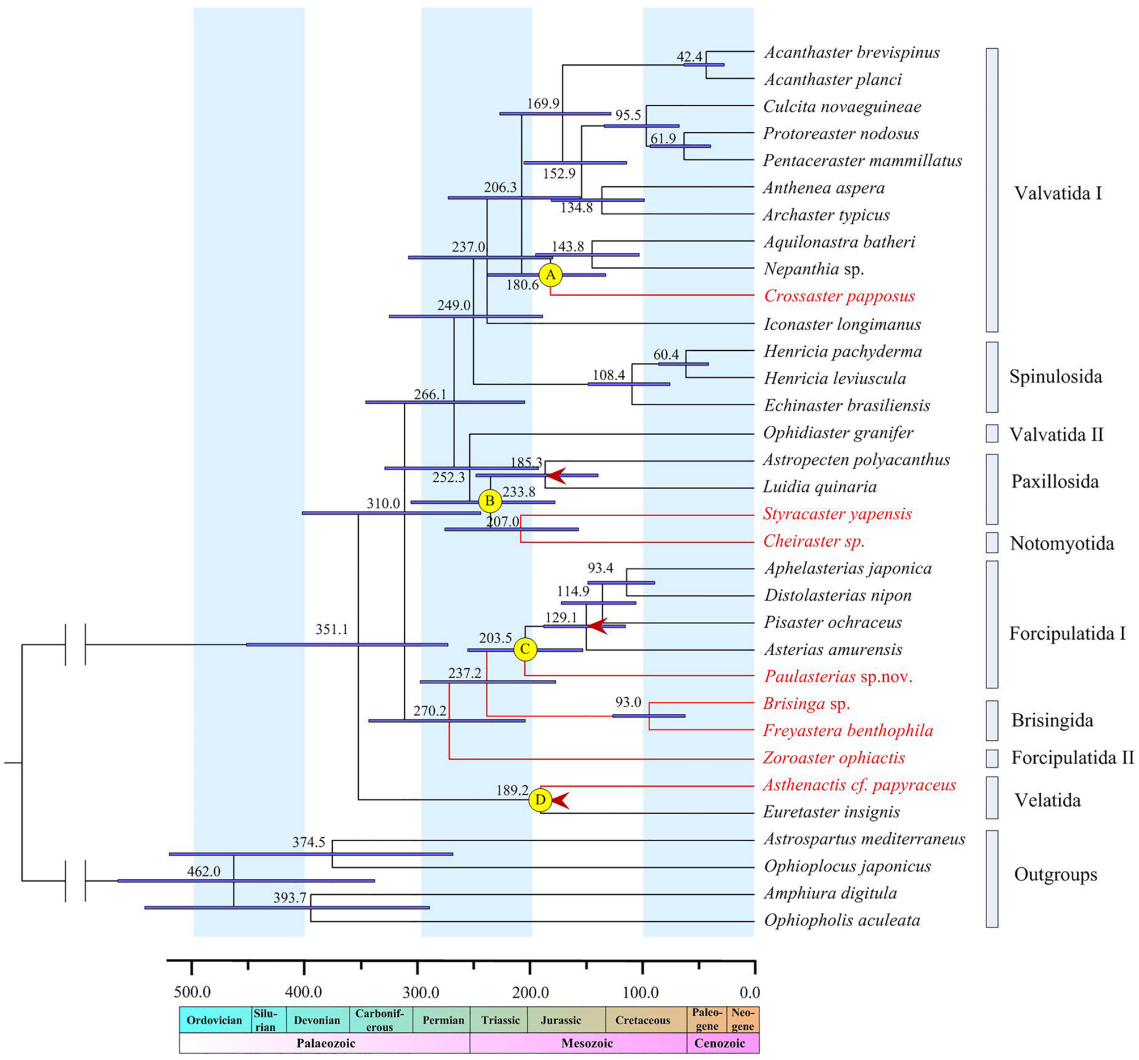
Time-calibrated mitogenomic phylogeny of Asteroidea estimated by the Bayesian relaxed-molecular clock method. The 95% HPD for each node were indicated in light purple bars. Calibrated nodes are indicated by red arrows. The divergence between the deep-sea and shallow-water species were marked by yellow circles (Nodel A, Nodel B, Nodel C and Nodel C).

Species of the class Asteroidea have an old fossil record, which can be dated back to the post-Paleozoic^15,76^. The fossil record and current accounts suggested that most of the asteroids diversification occurred during the Permian–Triassic transition interval^1,77^. Based on our time-calibrated phylogeny, the MRCA of Asteroidea originated around the boundary of Devonian and Carboniferous (351.1 Ma with 95% highest posterior density interval [HPD] 275.9–454.9 Ma), which supported the first important faunal shift of Paleozoic asteroid evolution the Late Devonian^78^. The diversification times of major lineages of Asteroidea are estimated as follows: Forcipulatida + Paulasteriidae + Brisingida, 270.2 Ma (95% HPD 208.2–347.1 Ma); Paxillosida + Notomyotida + Valvatacea II, 252.3 Ma (95% HPD 191.5–327.9 Ma); Valvatacea I, 237.0 Ma (95% HPD 181.5–310.4 Ma); Velatida, 189.2 Ma (95% HPD 179.5–198.8 Ma); Spinulosida, 108.4 Ma (95% HPD 74.3–147.5 Ma) (Fig. 5). These major lineages of Asteroidea likely originated around the boundary of Permian–Triassic (240–270 Ma). This result was consistent with second important faunal shift of the Paleozoic asteroid evolution, during the late Paleozoic to early Mesozoic transition to modem asteroids^78^. Twitchett and Oji^77^ suggested that the Permian–Triassic interval was a bottleneck in the evolutionary history of the echinoderms (including asteroids). Paleozoic lineages of asterozoans and early asteroids underwent extinction during this interval and was followed by re-diversification of at least one surviving lineage.

The deep-sea and shallow-water Valvatida species split at 180.6 Ma (95% HPD 108.9–199.7 Ma) (Node A). The deep-sea species of Paxillosida and Notomyotida diverged with its closest shallow-water relative at 233.8 Ma (95% HPD 176.7–304.6 Ma) (Node B). The divergence between the deep-sea species of Paulasteriidae and shallow-water Forcipulatida was located at 203.5 Ma (95% HPD 157.5–260.1 Ma) (Node C). The deep-sea and shallow-water species of Velatida split at 189.2 Ma (95% HPD 179.5–198.8 Ma) (Node D). The results indicated that the divergence between the deep-sea and shallow-water asteroids coincided approximately with the Triassic-Jurassic transition, which was marked by mass extinction event^79,80^. The widespread extinction created new opportunities for marine faunas, and contributed to the rapid divergence of both deep-sea and shallow water taxa.

### Positive selection analysis

We evaluated the potential positive selection pressures in deep-sea asteroids lineages, because colonisation may impact the function of mitochondrial oxidative phosphorylation genes. When the ω ratio for the 13 concatenated mitochondrial PCGs were compared between the deep-sea and shallow water sea stars, we failed to find a significant difference in their ω (Ks/Ks) ratios (chi-square: *p* > 0.05) (Table 2), implying that the ω ratio of deep-sea asteroids lineages (ω1 = 0.09158) have no significantly difference with the shallow water ones (ω0 = 0.09132). This may be biased by the sample sizes.

**Table 2:** Selective pressure analyses of the mitochondrial OXPHOS genes of asteroids.

Branch-specific models					
Models	lnL	Parameter estimates	Model compared	2ΔlnL	*p*-values
Free-ratio model (M1)	− 204017.5186		M1 versus M0	− 1823.43544**	0.0000
Two-ratio model (M2)	− 204929.2363	ω0 = 0.09129 ω1 = 0.09158	M2 versus M0	0.00565	0.9402
One-ratio model (M0)	− 204929.2391	ω = 0.09132			

Branch	Branch-sits models				
	Species	Gene	Positively selected sites		
Foreground-lineage I	*C. papposus*	*atp8*	45 K 0.966*		
		*nad2*	229 F 0.957*		
			279 G 0.960*		
		*nad6*	110 W 0.997**		
			156 S 0.973*		
Foreground-lineage II	*S. yapensis* + *Cheiraster* sp.	*nad2*	45 C 0.960*		
		*nad4*	375 K 0.957*		
Foreground-lineage III	*Paulasterias* sp.	*nad2*	158 G 0.974*		
			201 G 0.961*		
		*nad3*	20 A 0.970*		
		*nad4*	417 K 0.961*		
Foreground-lineage IV	*F. benthophila* + *Brisinga* sp.	*cox3*	145 A 0.979*		
		*cytb*	324 T 0.953*		
		*nad1*	88 C 0.962*		
			277 S 0.972*		
		*nad2*	42 P 0.958*		
			196 Y 0.965*		
			280 G 0.960*		
		*nad4*	11 T 0.976*		
			78 L 0.981*		
			101 L 0.984*		
			188 S 0.986*		
			254 S 0.983*		
			453 K 0.976*		
		*nad4l*	42 T 0.987*		
			81 S 0.989*		
Foreground-lineage V	*Z. ophiactis*	*nad2*	21 V 0.952*		
			146 V 0.972*		
			213 S 0.989*		
		*nad4l*	41 N 0.992**		
Foreground-lineage VI	*A. papyraceus*	*nad2*	281 L 0.957*		
		*nad4*	45 H 0.985*		
			362 L 0.982*		
			405 A 0.974*		

* 0.001 < p < 0.01 ** p < 0.001

It is the positive selection that is the basis of adaptation evolution^81^. Although the mitochondrial genes have been considered to be under purifying (negative) selection^56^, the signatures of positive selection may act on only a few sites in a gene sequence in some extreme environments^53,82–84^. In our results, BEB analysis of individual genes identified well-supported (PP ≥ 0.95) positively selected sites in two genes (*nad2*, *nad4*: total of 2 sites) along the *S. yapensis* + *Cheiraster* sp. lineage, three genes (*nad2*, *nad3*, *nad4*: total of 4 sites) along the *Paulasterias* sp. lineage, six genes (*cox3*, *cytb*, *nad1*, *nad2*, *nad4*, *nad4l*: total of 15 sites) along the *F. benthophila* + *Brisinga* sp. lineage, two genes (*nad2*, *nad4l*: total of 4 sites) along the *Z. ophiactis* lineage, and two genes (*nad2*, *nad4*: total of 4 sites) along the *A. papyraceus* lineage (Table 2). However, no positively selected sites were found along the *C. papposus* lineage. Although the positively selected genes vary for different deep-sea asteroids lineages, most of the positively selected loci were located in the NADH dehydrogenase (Complex I). Currently, the roles of NADH dehydrogenase in deep-sea adaptation still unclear, however, changes in these amino acid have functional implications and are a part of the adaptive response. Researchers have discovered that NADH dehydrogenase was the repeated target of positive selection in diverse deep-sea animals^53,82,83^. Under the extreme environment of deep sea, survival needs a modified and adapted energy metabolism^53,82,83^. As the first and largest enzyme complex, NADH dehydrogenase is supposed to act as proton-pumping devices in the mitochondrial OXPHOS process^57,85^. Therefore, mutations in the NADH dehydrogenase genes may effect metabolic efficiency^57,86^. So we predicted that the positively selected NADH dehydrogenase genes may play an important part in the adaptation of asteroids to deep-sea environment.

## Materials and methods

### Specimen collection and mitochondrial genome sequencing

Specimen collection information was shown in Table 1. All specimens were immediately preserved in 95% ethanol following collection. Total genomic DNA was extracted using the E.Z.N.A Tissue DNA kit (OMEGA, Wuhan, China) following manufacture’s protocols. The paired-end libraries were constructed using TruSeq Nano DNA Sample Prep Kit (Illumina, San Diego, CA, USA) with an insert size of 450 bp. The above libraries were sequenced by an Illumina (San Diego, CA, USA) HiSeq 4000 platform (2 × 150 bp paired-end reads).

### Mitochondrial genome assembly

The paired-end raw sequences for the five samples were trimmed using Trimmomatic 0.39^87^ with the following parameters: ILLUMINACLIP:TruSeq3-PE.fa:2:30:10 LEADING:3 TRAILING:3 SLIDINGWINDOW:4:15 MINLEN:75. The obtained clean reads were assembled de novo by NOVOPlasty software^88^ with default parameters. In the seed extension algorithm achieved via NOVOPlasty, the *cox1* or 16S rDNA gene fragment of their closed related species (*cox1* of *C. dawsoni* (JQ918233) for *Cheiraster* sp., 16S rDNA of *Paulasterias* sp. AAR (KT258987) for *Paulasterias* sp., *cox1* of *Asthenactis* sp. CLM-2012 (JQ918238) for *A. papyraceus*, *cox1* of *Z. spinulosus* (JQ918246) for *Z. ophiactis*, and *cox1* of *Brisinga* sp. 2 RSIOAS023 (MN885902) for *Brisinga* sp.) was used as a seed sequence. The mtDNA will be circularized when the length is in the expected range and both ends overlap by at least 200 bp. Considering the influence of the nuclear mitochondrial DNA (NUMT) sequences^89^, we checked the obtained mitochondrial genome sequences completely, as significantly higher coverage may pinpoint to sequences derived from nuclear DNA or duplicated regions. The evenness and similarity for the coverage of each individual gene and control region (Supplementary Table S4) mean that the NUMT sequences may not confound the results from the Illumina sequencing. The control region was usually considered as “difficult genomic regions”. The fragments located in the large non-coding regions, were also verified by PCR amplification and Sanger sequencing^90^. The PCR Primer sequences and positions were shown in Supplementary Table S5.

### Mitochondrial genome sequence annotation and analysis

MITOS web server^91^ was preliminarily used to annotate the PCGs, 2 rRNAs and tRNAs under default settings and the invertebrate mitochondrial genetic code. Boundaries of the PCGs and rRNAs genes were determined by comparing them with the homologous genes of other asteroids. The tRNA genes and their secondary structures were further confirmed by the ARWEN 1.2.3.c^92^. The gene orders of asteroids were compared with that of the echinoderm ground pattern. The CREx webserver (http://pacosy.informatik.uni-leipzig.de/crex/)^93^ was used to compare the mitochondrial gene order and deduce the gene rearrangement scenarios based on common intervals. The arrangement of all the genes (PCGs, rRNAs and tRNAs) were considered. The nucleotide composition and codon usage counts were computed by MEGA 5^94^ based on the invertebrate mitochondrial genetic code (genetic code = 5). The differences and significant levels (*p*-value) for nucleotide composition and amino acid contents between the deep-sea species and the asteroids from shallow waters were performed by a two-tailed *t-test* and the chi-square test in IBM SPSS Statistics, release 19.0.0.1, respectively. The complete mtDNA sequences have been deposited in the GenBank database with the accession numbers MZ702701-MZ702705.

### Phylogenetic inference

Thirty-three asteroids mitogenomes including the five new sequenced in this study were used for phylogenetic analysis, with four species of ophiuroids used as outgroups. The detailed informations of these available sequences were listed in Supplementary Table S6. In the following study, the species collected below 200 m are treated herein as “deep-sea group”, and these from 0 to 200 m are considered as “shallow water group”. The nucleotide and amino acid sequences from each of the 13 PCGs were aligned separately using MAFFT^95^ with default settings. The ambiguously aligned and variable regions were eliminated using the program Gblocks^96^ with default stringent parameters. Then, the aligned homologous nucleotide and amino acid sequences were concatenated into a single supermatrix with FASconCAT^97^. The best-fit substitution models for each nucleotide and amino acid partition were selected by PartitionFinder^98^.

Phylogenetic analysis was carried out using maximum likelihood (ML) and Bayesian inference (BI). ML analysis was performed in IQ-TREE web server^99^ with partition models. The node reliability was assessed using 1000 ultrafast bootstrap replicates^100^. BI analysis was employed using MrBayes 3.1 software^101^ with partition models. A total of 10,000,000 generations (Markov chain Monte Carlo, MCMC) were run, with a sampling frequency of 1,000 generations to allow adequate time for convergence. The run was stopped when the standard deviation of split frequencies was less than 0.01. The software Tracer v1.6^102^ was used to checked the parameters of the two independent runs (effective sampling size for all parameters > 200). The first 5000 trees were burnin. The 50% majority rule consensus tree was constructed using the remaining 5000 sampled trees.

### Ancestral geographic area and habitat reconstructions

The ancestral geographic area and habitat of the asteroids were reconstructed by RASP v. 3.2^103^. The RASP provides a graphical user interface (GUI) to specify a phylogenetic tree and geographic distributional information, draws pie charts on the nodes of a phylogenetic tree to indicate levels of uncertainty, and generates high-quality exportable graphical results to reconstruct ancestral geographical distributions. Three models, i.e., Statistical Dispersal-Vicariance Analysis (S-DIVA), Statistical Dispersal-Extinction-Cladogenesis (S-DEC) and Dispersal-Extinction-Cladogenesis (DEC) were used. In the model S-DIVA, the occurrence of an ancestral range at a node was calculated by the frequency of all of the alternative reconstructions generated by the DIVA algorithm for each tree in the data set. The DEC and S-DEC models export the likelihood of all possible biogeographic scenarios estimated at a given node. Akaike weights were then calculated and interpreted as the relative probability of different ancestral ranges, which were displayed as pie charts on the nodes of a phylogenetic tree.

The mtDNA of *Pisaster ochraceus* was submitted directly in NCBI with no specimen collection information. So the location of the species was equivocal, which was not used in this analysis. Distribution data were compiled from the specimen collection records (Supplementary Table S6) and from the summary of Mah and Blake^1^. For the ancestral geographic area reconstruction, four large-scale biogeographic patterns were coded as follows: (A) Tropical Western Atlantic, (B) Temperate Western Pacific, (C) Tropical Western Pacific Ocean, and (D) Arctic Ocean. For the ancestral habitat reconstruction, the asteroids were categorized into (A) tropical, (B) temperate, and (C) cold.

### Divergence time estimation

BEAST v2.3.3^104^ estimated the divergence times of major clades of Asteroidea using the following parameters: the uncorrelated relaxed lognormal clocks, random starting trees and a the Yule process. The divergence times were sampled once every 1,000 generations from 10 million MCMC generations. Tracer v1.6^102^ examined the effective sample sizes (ESS) of parameters (ESS > 200). The initial 50% of cycles were burn-in. TreeAnnotator (BEAST software) summarized the 95% highest posterior densities (HPD) and the maximum-clade-credibility tree topology with median ages. FigTree 1.4^105^ was used to view the the tree, divergence times and 95% HPD around the node ages. The divergence times were calibrated with three fossil taxa. The age of order Velatida was constrained with a lognormal distribution between 182.0 and 201.6 Ma based on *Plesiastropecten hallovensis*^106,107^. The age of family Astropectinidae was constrained with a lognormal distribution between 161.2 and 171.6 Ma^108^ based on *Tethyaster jurassicus*. The age of family Goniasteridae was constrained between 167.7 and 171.6 Ma, based on *Comptoniaster sharpii*^109^.

### Positive selection analysis

The codon-based likelihood approach implemented in the CODEML program of PAMLx package^110^ was used to evaluate the changes in selective pressure between the deep-sea asteroids and other shallow water ones. The ML and BI tree topology inferred in this study was used. Non-synonymous to synonymous substitution rates (Ka/Ks, ω) was considered as the measure of selective pressure at the protein level.

The branch-specific model were used to test if the selective pressure between the deep-sea and shallow water starfish were different. For branch-specific model, the comparison between “free-ratio” (M1) and “one-ratio” models (M0), “two-ratio” (M2) and “one-ratio” models (M0) were conducted in the combined dataset of 13 protein-coding genes using the likelihood ratio test (LRT). The Chi-square test^111^ was applied for testing *p*-values.

The branch-site model was used to detect the sites under positive selection in each mitochondrial gene of the foreground-lineages (deep-sea asteroids lineages), which allowed ω to vary across branches and sites. For this model, the asteroids phylogeny was partitioned into foreground and background lineages in multiple ways. As the branch-site model does not allow for multiple foreground lineages, we considered the branches leading to the deep-sea asteroids as foreground-lineage separately. Firstly, the lineage of *C. papposus* was set as the foreground-lineage I. Secondly, we set the monophyletic branch leading to *S. yapensis* and *Cheiraster* sp. as the foreground-lineage II. Thirdly, we consider the branch *Paulasterias* sp. as the foreground-lineage III. Fourthly, the monophyletic branch leading to *F. benthophila* and *Brisinga* sp. was set as the foreground-lineage IV. Fifthly, the branch leading to *Z. ophiactis* was set as the foreground-lineage V. Lastly, the branch leading to *A. papyraceus* was set as the foreground-lineage VI. LRT were conducted to test if the alternative model (MA) fits the data significantly better than the corresponding null model (MA0). All the positively selected sites were determined by Bayes empirical Bayes (BEB) method^112^ with posterior probabilities of ≥ 0.95.

## Supplementary Information

Supplementary Legends.

Supplementary Table S1.

Supplementary Table S2.

Supplementary Table S3.

Supplementary Table S4.

Supplementary Table S5.

Supplementary Table S6.

## Data Availability

All relevant data are within the manuscript and its Supporting information files.
