# Supplementary material for: Mitogenomics provides new insights into the phylogenetic relationships and evolutionary history of deep-sea sea stars (Asteroidea)
Source: Sci Rep. 2022 Mar 18;12:4656. doi: 10.1038/s41598-022-08644-9 (PMC8933410; doi:10.1038/s41598-022-08644-9)
Supplement: Supplementary file 1 — Supplementary Legends. [file 41598_2022_8644_MOESM1_ESM.docx]

**Supporting information**

**Supplementary Table S1**. Organization of the mitochondrial genomes of *Cheiraster* sp., *Paulasterias* sp., *Asthenactis papyraceus*, *Zoroaster ophiactis* and *Brisinga* sp.

**Supplementary Table S2**. Base composition of mtDNA in Asteroidea.

**Supplementary Table S3**. The proportions (%) of amino acid of mitochondrial PCGs from Asteroidea.

**Supplementary Table S4**. The coverage for each individual gene and control region.

**Supplementary Table S5**. Primers used for amplification of control region in sea star mitochondrial genome.

**Supplementary Table S6**. List of samples included in the analyses in this study.
